# Sensory regulation of meal sorting in *Aedes aegypti* mosquitoes

**DOI:** 10.1038/s41598-024-83172-2

**Published:** 2024-12-30

**Authors:** Emi Maekawa, Anupama A. Dahanukar

**Affiliations:** 1 Department of Molecular, Cell and Systems Biology, University of California, Riverside, CA 92521 USA; 2 Present Address: Department of Tropical Medicine, The Jikei University School of Medicine, Tokyo, 105-8461 Japan

**Keywords:** Mosquito, Meal destination, Feeding, Crop, Midgut, Blood, Feeding behaviour, Gustatory system, Sensory processing

## Abstract

Meal sorting in mosquitoes is a phenomenon whereby ingested blood and sugar meals are directed to different destinations in the alimentary canal. We undertake a systematic analysis and show that entry of blood in the midgut is influenced by blood components, temperature, and feeding mode, while sugar solutions are directed to the crop in a dose-dependent manner. Sweet and nutritive sugars, like sucrose and maltose, enter the crop more efficiently compared to non-sweet or non-nutritive sugars. Additionally, the robustness of meal sorting declines with mosquito age and is compromised in mutants of candidate thermoreceptors. Proper blood meal sorting is crucial for optimal egg production, as disruption of this process by adding sucrose results in reduced fecundity. Furthermore, certain amino acids essential for vitellogenesis are preferentially directed to the midgut. Our findings provide new insights into the meal sorting mechanism, with implications for mosquito reproduction and vectorial capacity.

## Introduction

Mosquitoes are vectors of numerous human and animal pathogens of medical and veterinary significance, causing millions of infections and deaths annually. Adult mosquitoes of both sexes rely on nectar and honeydew as energy-rich sugary meals, utilizing ingested sugar for metabolic processes vital for flight, survival, and reproduction^1–3^. However, blood-feeding is essential for egg development in anautogenous mosquito species, with females requiring at least one blood meal during each gonotrophic cycle to obtain necessary nutrients^4^. This need for blood leads to frequent host interactions, making anautogenous mosquitoes among the most effective disease vectors.

Mosquito-borne diseases are spread to 700 million people every year, resulting in more than one million deaths^5^. In 2023, the incidence of dengue reached unprecedented levels. The annual burden of dengue infections is estimated to be > 100 million cases per year in over 125 countries^6,7^.

Hematophagous dipterans, such as mosquitoes and sand flies, possess a distensible midgut and direct ingested blood to the midgut for digestion and sugar to the crop for temporary storage, a phenomenon termed “meal sorting” or “meal switching”^8–13^. This adaptation provides a remarkably effective foraging strategy, allowing these insects to take advantage of both plant and animal food sources as they become available^10,12^. Additionally, the storage of sugar solutions in impermeable structures may provide a water reservoir that can be transferred to the midgut for absorption as needed^14^, thereby preventing rapid drops in hemolymph osmotic pressure and protecting the midgut epithelium or other tissues from osmotic stress^1,15–17^.

A robust meal switching system is intertwined with specialized digestive functions. The crop and midgut regions are distinct in terms of gene expression. Transcriptomic atlases in *Aedes aegypti* delineate five distinct gut regions (crop, proventriculus, anterior midgut, posterior midgut, and hindgut)^18^, and highlight the specialized roles of the crop and midgut in terms of meal storage and digestion. While the crop’s primary function is to store and gradually release sugary meals, the midgut is adapted for efficient blood meal digestion and nutrient absorption^19^. Reflecting these differences, the midgut shows high enrichment of genes involved in digestion and absorption of nutrients, and removal of toxic byproducts of blood digestion^18^. Furthermore, the separation of sugar and blood meals through meal switching may help prevent the potential inhibition of blood digestion by plant-derived compounds, such as trypsin inhibitors, that could be present in sugar meals^1,20,21^.

The crop and midgut also harbor bacterial populations that differ in composition, function, and abundance^22^ and the gut microbiome plays a crucial role in blood digestion^23^ and impacts the mosquito’s longevity and fecundity^24^. Furthermore, immune-related genes are also differentially expressed in the crop and midgut regions^18^. Enhanced immune responses in the proventriculus and anterior midgut may serve as a barrier to blood-borne pathogens. Mosquito susceptibility to infection by human pathogens is also impacted by the gut microbiome^25–27^. Thus, the separate abilities of the crop and midgut are crucial for effective utilization of sugar and blood meals and also have implications for the mosquito’s response to blood-borne pathogens.

Despite the importance of meal switching, the underlying mechanisms remain poorly understood. Proper function of both the crop (diverticular) and midgut (cardiac) sphincters is likely essential. A recent study implicated a gut-specific microRNA miR-275 in cardiac sphincter function and blood meal digestion^28,29^, highlighting the importance of proper valve function in meal distribution and optimal egg production. However, the cues that regulate the valves are not clear. The involvement of receptors for sensing glucose and blood in mosquitoes has been proposed^20,30^, with specific sugars and blood components stimulating pit organs and papillary sense organs, respectively, leading to the relaxation of diverticular and cardiac sphincters^30^. Combinations of physical factors (temperature and osmotic pressure), chemical phagostimulants and nutrients (ATP and ADP, sugars, amino acids), mouthpart deployment, and the sequence of stimuli prior to feeding^8,10,11,30–35^ have all been implicated in meal switching from studies of various hematophagous dipterans. However, the studies represent a diversity of strains, methodologies, and experimental conditions^36–38^, and a unified explanation of the underlying mechanisms has not emerged. Moreover, this problem has not been revisited in the past couple of decades with modern discovery approaches.

Here, we systematically re-evaluate the factors that influence the destination of ingested meals in female *Ae. aegypti* mosquitoes. We find that blood meals are actively directed to the midgut, bypassing the crop, and this process is influenced by blood components (especially leukocytes, platelets, and red blood cells), temperature, and feeding mode (i.e., skin piercing). The robustness of this switching mechanism may be compromised with aging and in mutants of candidate thermoreceptors (*AaegTRPA1* and *AaegGr19*). In contrast, sugar solutions are diverted to the crop in a dose-dependent manner, with sweet and nutritive sugars (e.g., sucrose, maltose, D-glucose) being more efficiently directed to the crop compared to non-sweet or non-nutritive sugars. Temperature and feeding mode cues associated with blood-feeding can shift the destination of water and low-sugar meals towards the midgut. Disrupting the proper sorting of blood meals by adding sucrose results in reduced egg production and follicle size, highlighting the functional importance of meal sorting. Lastly, we observe that the entry of amino acids into the crop depends on their identity, with some amino acids essential for vitellogenesis^4^ (e.g., leucine and tryptophan) being preferentially directed to the midgut. Overall, our findings reveal a complex interplay of sensory cues in regulating meal destination in mosquitoes, which has important implications for their reproductive success and vectorial capacity.

## Results

### Blood enters the midgut rather than the crop during blood-sucking mode

**Fig. 1:**
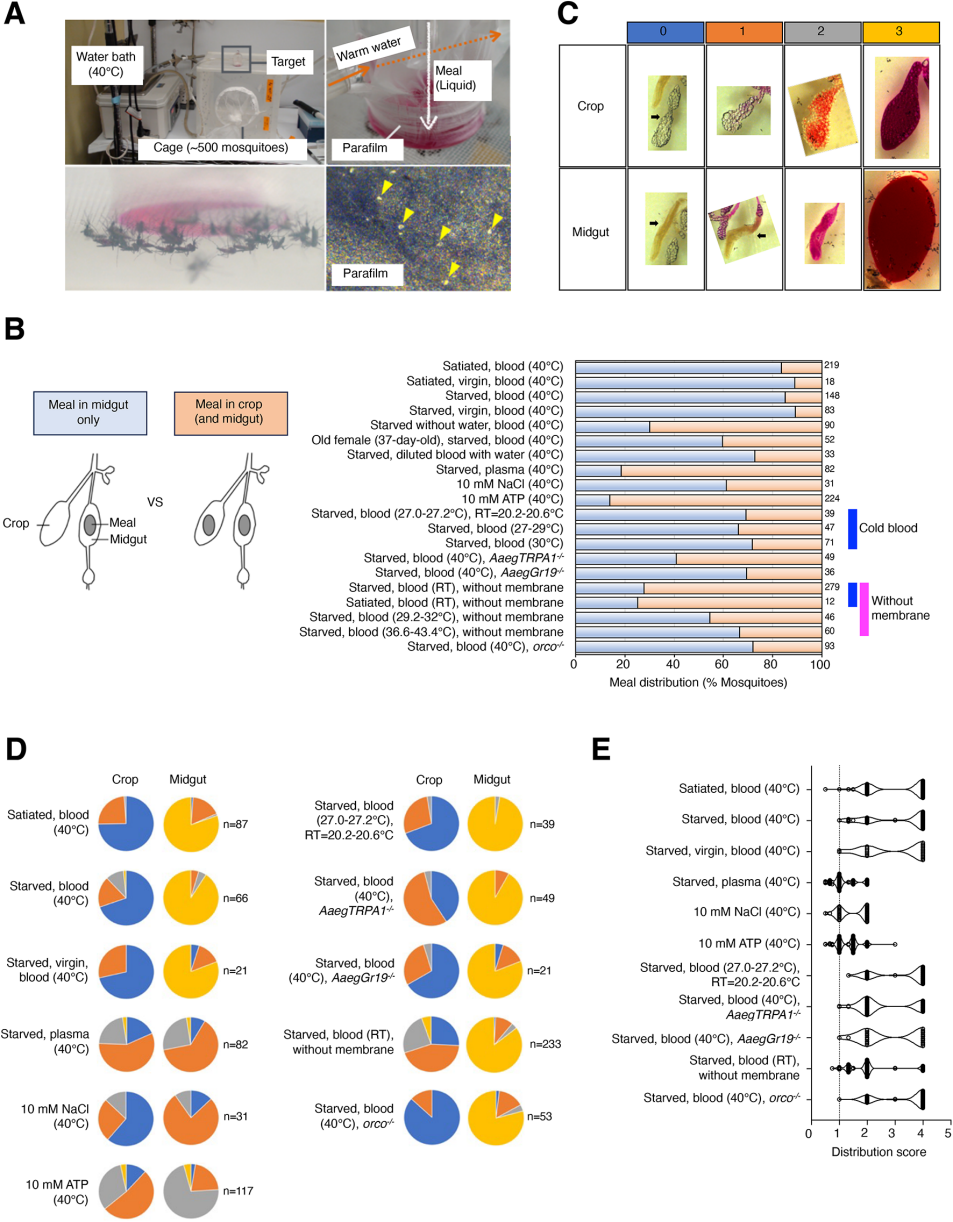
Blood preferentially enters the midguts during blood-sucking mode. (**A**) Photograph of the artificial membrane feeding system (top left) and an enlarged view of the feeder cup in which the meal solution is heated with circulating warm water and the bottom is covered with stretched parafilm (top right). Mosquitoes land on the membrane (bottom left) and pierce it to feed, and perforations can be seen in the stretched membrane (yellow arrowheads, bottom right). (**B**) Diagram showing how meal destination patterns were scored (left) and percent of mosquitoes showing “meal in midgut only” (light blue) and “meal in crop (and midgut)” (light orange) when tested under the indicated feeding conditions. Numbers of fed mosquitoes for each condition are indicated to the right of the graph. Unless otherwise specified, RT = 28 °C. (**C**) Photographs of dissected guts showing imbibed solutions in the crop and midgut scored from 0 (none) to 3 (highest). The scores are color coded as follows: 0 = blue, 1 = orange, 2 = gray, and 3 = yellow. (**D**) Pie charts showing fractions of mosquitoes scored as in C for feeding conditions indicated on the left. Total numbers of mosquitoes for each feeding condition are noted on the right. (**E**) Distribution scores (see STAR METHODS) for results shown in D.

We examined the destination of blood meals ingested by female mosquitoes, by feeding mosquitoes aged 5 to 14 days old with blood heated to approximately 40 °C in an artificial membrane feeder and dissecting the mosquitoes after feeding (Fig. 1A). The presence of blood in the crop and midgut was assessed visually under a stereomicroscope (Fig. 1B) and the amount present in each organ was scored on a scale ranging from 0 (none) to 3 (engorged) (Fig. 1C). If blood was not detected in either the crop or midgut, the individual was excluded from the analysis as unfed. For simplicity, dorsal diverticula were ignored. To quantify the relative distribution of the meal in the midgut and crop, we calculated a distribution score for each mosquito (see STAR METHODS). A score of 1 would indicate equal proportions of the meal in the midgut and crop, whereas scores > 1 would indicate a higher proportion of the meal in the midgut. In most fed mosquitoes (85.1–89.2%), blood was present only in the midgut when mosquitoes were wet starved for ~ 24 h, but the blood meal destination was drastically disrupted when mosquitoes were starved without water for ~ 24 h (Fig. 1B; starved without water, blood (40 °C)). For all subsequent experiments, therefore, we allowed free access to water during the starvation period. Regardless of mating (virgin or mated) or fed status (wet starved for ~ 24 h or sugar-fed) most fed mosquitoes had the blood meal only in the midgut (Fig. 1B; satiated, blood (40 °C); satiated, virgin, blood (40 °C); starved, blood (40 °C); starved, virgin, blood (40 °C); and 1E; satiated, blood (40 °C); starved blood (40 °C); starved, virgin, blood (40 °C), [mean distribution score: 3.048–3.179]). For the fraction of females in which we observed blood in the crop, the amount was almost negligible (Fig. 1D; satiated, blood (40 °C); starved, blood (40 °C); starved, virgin, blood (40 °C)). Together with previous studies, these results are consistent with the idea that the blood meal bypasses the crop, whose valve is closed, and is actively pumped into the midgut. In 37-day-old mosquitoes, the fraction of animals in which blood was found solely in the midgut fell to 59.6%, indicating that the robustness of the switching mechanism may be compromised with aging (Fig. 1B; old female (37-day-old), starved, blood (40 °C)).

To test the effect of blood components on bypassing the crop during ingestion, we first fed mosquitoes with blood that was diluted 1:1 with water. Although a majority of mosquitoes that fed on diluted blood (72.7%) exhibited a “midgut only” distribution of the meal (Fig. 1B; starved, diluted blood with water (40 °C)), the proportion was lower than that found for mosquitoes fed on undiluted blood, suggesting that a feature of the undiluted blood is important for the switching mechanism. We next tested plasma, which was separated from cellular components by centrifugation (1200 x g). Plasma alone elicited a drastic reduction in intake; furthermore, the meal destination was restricted to the midgut in only 18.3% of the fed individuals (Fig. 1B; starved, plasma (40 °C), and 1D; starved, plasma (40 °C), and 1E; starved, plasma (40 °C) [mean distribution score: 1.165]). These results indicate that the presence of sufficient amounts of blood components, especially leukocytes, platelets, and red blood cells, is important for diverting the blood meal to the midgut.

Two blood components, NaCl and ATP, are known to stimulate blood-feeding behavior^39,40^. We therefore tested the meal destination of each of these components. In many individuals, the ingested 10 mM NaCl solution was found only the midgut (61.3%) (Fig. 1B; 10 mM NaCl (40 °C), and 1E; 10 mM NaCl (40 °C) [mean distribution score: 1.489]), although the ingested volume was relatively small (Fig. 1D; 10 mM NaCl (40 °C)). This result is consistent with a previous study, which reported that a solution of 150 mM NaCl entered the midgut rather than the crop^39^. In contrast, although a large amount of 10 mM ATP solution entered the midgut (Fig. 1D; 10 mM ATP (40 °C)), the crop also contained the solution (Fig. 1B; 10 mM ATP (40 °C), and 1E; 10 mM ATP (40 °C) [mean distribution score: 1.317]). Thus, NaCl in the plasma may serve as a cue for proper blood meal destination. On the other hand, as the plasma contains not only NaCl but various other factors including proteins, sugars, and amino acids, the action of the NaCl may have been masked (Fig. 1B; starved, plasma (40 °C)).

We next assessed the impact of blood temperature on the switching mechanism. We reduced the blood temperature from ~ 40 °C (default) to a range between 27 °C and 30 °C. To increase the difference between ambient and blood temperature, the experiments were carried out at a lower room temperature (20.2–20.6 °C as compared to the default 28 °C). With blood at 27–30 °C, the proportion of mosquitoes exhibiting the “midgut only” phenotype decreased to 66.0-71.8% (Fig. 1B; starved, blood (27.0–27.2 °C), RT = 20.2–20.6 °C ; starved, blood (27–29 °C); starved, blood (30 °C), and 1E; starved, blood (27.0–27.2 °C), RT = 20.2–20.6 °C [mean distribution score: 3.342]), although the volumes in the midgut were similar to those found with warm blood (Fig. 1D; starved blood (27.0–27.2 °C), RT = 20.2–20.6 °C). To further evaluate the role of thermosensation in this process, we tested mutants for candidate thermoreceptors^41^, *AaegTRPA1* and *AaegGr19*. Trans-heterozygous *AaegTRPA1*^*−/−*^ and *AaegGr19*^*−/−*^ female mosquitoes were wet-starved for ~ 24 h and given warm blood. Notably, in both mutants, the amount of blood entering the midgut was comparable to wild type (Fig. 1D; starved, blood (40 °C), *AaegTRPA1*^*−/−*^ and 1D; starved, blood (40 °C), *AaegGr19*^*−/−*^ ), but the percentage of mosquitoes displaying the “midgut only” phenotype was reduced – 40.8% in *AaegTRPA1*^*−/−*^ and 69.4% in *AaegGr19*^*−/−*^ mutants (Fig. 1B; starved, blood (40 °C), *AaegTRPA1*^*−/−*^, 1B; starved, blood (40 °C), *AaegGr19*^*−/−*^, 1E; starved, blood (40 °C), *AaegTRPA1*^*−/−*^, and 1E; starved, blood (40 °C), *AaegGr19*^*−/****−***^ [mean distribution score: 2.667–2.873]). These results support the idea that temperature sensation plays a role in regulating blood meal destination.

In the artificial blood-feeding apparatus, a mosquito utilizes her stylet to puncture a stretched parafilm membrane, simulating host skin, to access blood in the feeder cup (Fig. 1A). To test whether the action of “piercing” informs meal destination we supplied blood via tissues or cotton balls saturated with blood, which we tested at three different temperatures. With the blood-soaked cotton ball at room temperature, the percentage of fed individuals exhibiting a “midgut only” phenotype was dramatically reduced to 27.6% (Fig. 1B; starved, blood (RT), without membrane and 1E; starved, blood (RT), without membrane [mean distribution score: 2.078]). Importantly, the ingested volume was nearly identical to that obtained with the membrane feeder (Fig. 1D; starved, blood (RT), without membrane). The disruption in blood meal destination was independent of starvation; sugar-fed females given blood-soaked cotton balls at room temperature exhibited a similar phenotype (Fig. 1B; satiated, blood (RT), without membrane). Interestingly, the proportion of individuals with a “midgut only” recovered to some extent (66.7%) when the blood temperature was elevated (Fig. 1B; starved, blood (29.2–32 °C), without membrane and 1B; starved, blood (36.6–43.4 °C), without membrane). Although there are differing observations about the role of *orco*-expressing neurons in the stylet in blood-feeding^42,43^, we next tested whether *orco*^−/−^ mutants exhibited deficits in meal sorting. The proportion of individuals with a “midgut only” was 72.04% (compared to 85.14% for wild type) and ingested volume was normal (Fig. 1B; starved, blood (40 °C), *orco*^*−/−*^, 1D; starved, blood (40 °C), *orco*^*−/−*^, and 1E; starved, blood (40 °C), *orco*^*−/−*^ [mean distribution score: 3.34]), indicating that *orco* may also have a partial impact on switching. Collectively, our findings suggest that the robustness of blood meal destination, wherein the crop valve is closed and blood enters the midgut exclusively, is influenced not only by the presence of blood components but also by temperature and feeding mode (i.e. skin piercing) prior to ingestion.

### Sucrose solutions are directed to the crop in a dose-dependent manner

Nectar represents a significant nutritional source for adult mosquitoes of both sexes^1^. Sucrose is a predominant component of numerous floral nectars and serves as a standard sugar for rearing mosquitoes in the laboratory. Sucrose concentrations in nectar are reported to range from 10 to 70% (~ 300–2000 mM)^44,45^. Previous work has shown that sugar solutions, ingested from soaked cellucotton or through a membrane, enter the crop^8^. Highly concentrated sugar solutions are also directed to the crop in non-hematophagous dipteran blowflies. To better understand the parameters that influence meal sorting, we wished to further characterize the destination of ingested sugar meals and determine how the factors such as sugar concentration, temperature and feeding mode influence the process. Animals were wet-starved for ~ 24 h prior to testing. Pink dye was added to the test solutions to track the meal destination.

**Fig. 2:**
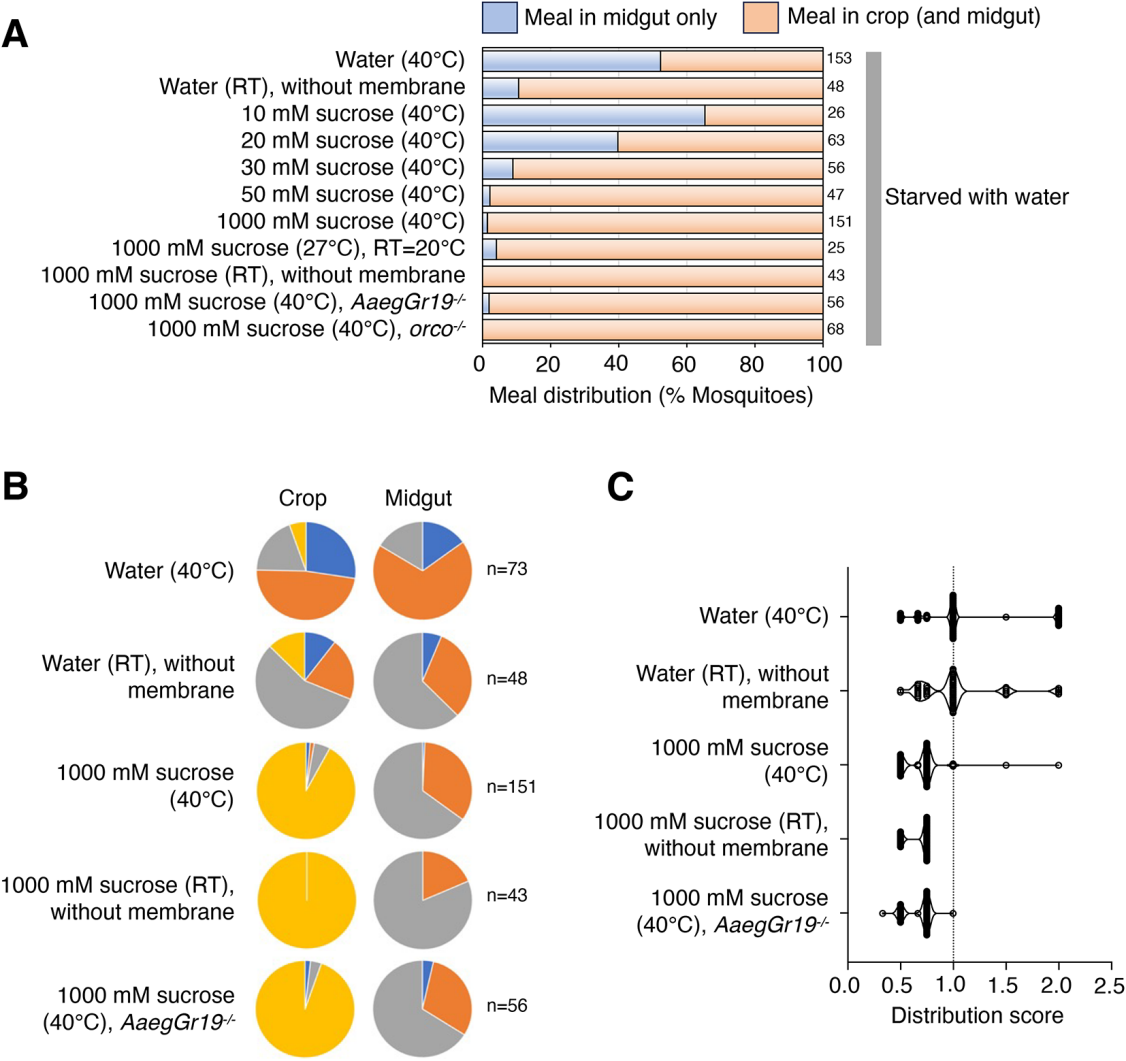
Sucrose solutions enter the crop in a dose-dependent manner. (**A**) Percent of mosquitoes showing “meal in midgut only” (light blue) and “meal in crop (and midgut)” (light orange) under indicated feeding conditions. For all experiments, mosquitoes were starved for ~ 24 h with access to water prior to feeding. Note that the following solutions were given in a membrane feeder heated to indicated temperature: water (40 °C); 10 mM-1000 mM sucrose (40 °C); 1000 mM sucrose (27 °C), RT = 20 °C; 1000 mM sucrose (40 °C), *AaegGr19*^*−/−*^, and 1000 mM sucrose (40 °C), *orco*^*−/−*^. Numbers of fed mosquitoes for each condition are indicated to the right of the graph. Unless otherwise specified, RT = 28 °C. (**B**) Pie charts showing fractions of mosquitoes from A scored and color coded as in Fig. 1. (**C**) Distribution scores (see STAR METHODS) for results shown in B.

When provided water control via an artificial membrane feeder (with membrane, with heat), about half of the females who ingested water exhibited a “midgut-only” phenotype (Fig. 2A; water (40 °C), 2B; water (40 °C), and 2C; water (40 °C) [mean distribution score: 1.132]). This is in contrast to the results obtained with water at room temperature provided via saturated cotton balls, in which case few individuals (< 10.5%) had the imbibed water restricted to the midgut (Fig. 2A; water (RT), without membrane, 2B; water (RT), without membrane, and 2C; water (RT), without membrane [mean distribution score: 1.028]). Given these observations, we performed follow up experiments using the artificial membrane feeder, which afforded the opportunity to test how various sugar solutions altered meal sorting in either direction. Low concentrations of sugar engendered a similar outcome (65.4%) as water (Fig. 2A; 10 mM sucrose (40 °C)), suggesting that in the presence of cues that are typically associated with blood-feeding (i.e. temperature and piercing mode) water and low-sugar meals shift towards a blood-meal-like distribution (i.e. meal in midgut only) in the gut.

With increasing concentrations of sugar, however, nearly all fed animals showed that the imbibed meal was diverted to the crop (Fig. 2A; 20-1000 mM sucrose (40 °C), 2B; 1000 mM sucrose (40 °C), and 2C; 1000 mM sucrose (40 °C) [mean distribution score: 0.6937]). Consistent with the notion that temperature and feeding mode are superfluous in controlling destination of high sugar meals, similar fractions of animals with crop meals were found for 1000 mM sucrose solutions given at a lower temperature (Fig. 2A; 1000 mM sucrose (27 °C), RT = 20 °C ) or without the feeding membrane (Fig. 2A; 1000 mM sucrose (RT), without membrane, and 2B; 1000 mM sucrose (RT), without membrane, and 2 C; 1000 mM sucrose (RT), without membrane [mean distribution score: 0.6830]). Moreover, high sugar solutions were normally diverted to the crop in *AaegGr19*^−/−^ and *orco*^*−/−*^ mutants (Fig. 2A; 1000 mM sucrose (40 °C), *AaegGr19*^*−/−*^, 2A; 1000 mM sucrose (40 °C), *orco*^*−/−*^, 2B; 1000 mM sucrose (40 °C), *AaegGr19*^*−/−*^, and 2 C; 1000 mM sucrose (40 °C), *AaegGr19*^*−/−*^ [mean distribution score: 0.6741]).

### Crop entry occurs with sweet and nutritive sugar solutions

**Fig. 3:**
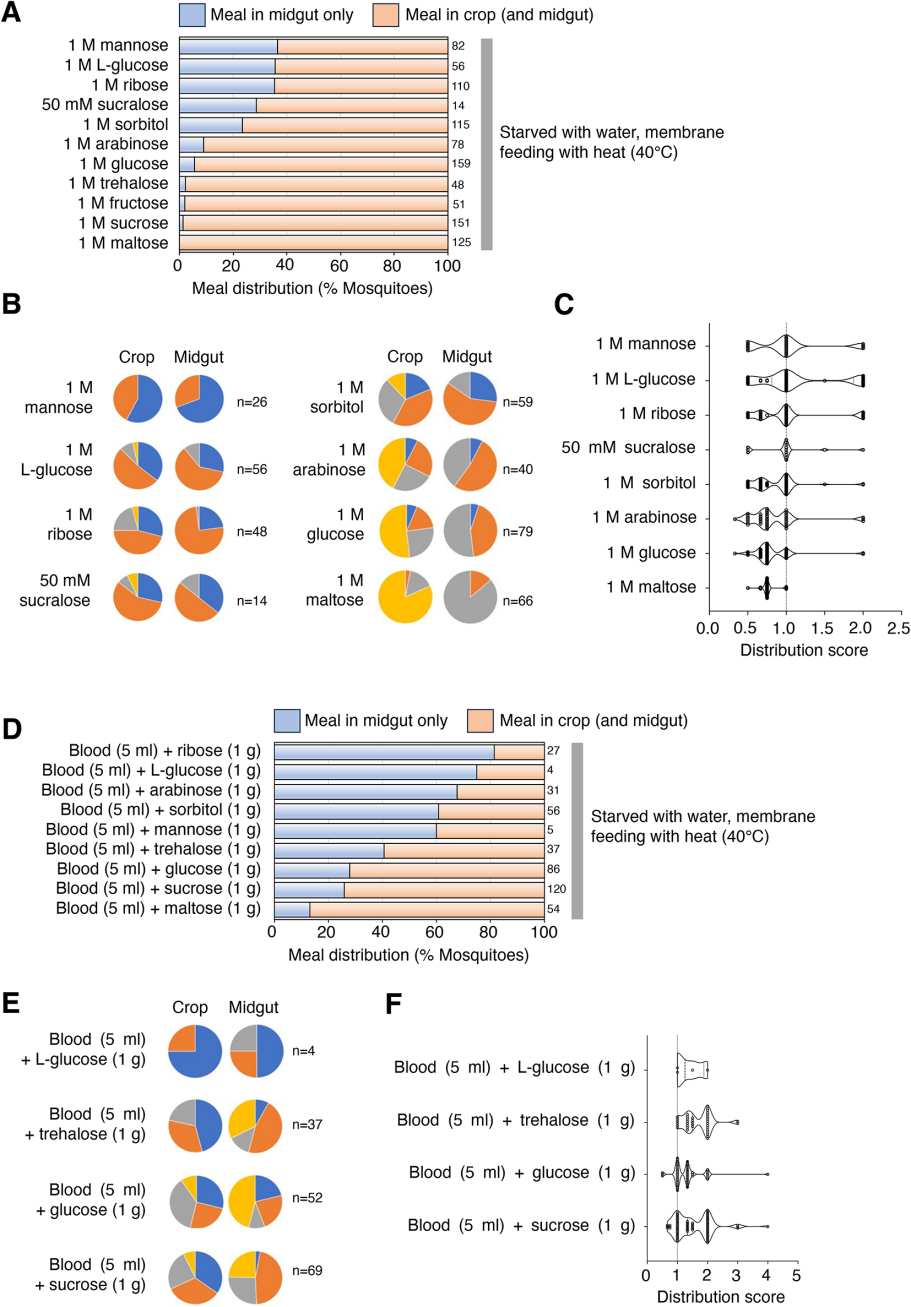
Sweet and nutritive sugar solutions tend to enter the crop. (**A**) Percent of mosquitoes showing “meal in midgut only” (light blue) and “meal in crop (and midgut)” (light orange) under indicated feeding conditions. Numbers of fed mosquitoes for each condition are indicated to the right of the graph. Data in 1 M sucrose is from Fig. 2A. (**B**) Pie charts showing fractions of mosquitoes from A scored and color coded as in Fig. 1. (**C**) Distribution scores (see STAR METHODS) for results shown in B. (**D**) Percent of mosquitoes showing “meal in midgut only” (light blue) and “meal in crop (and midgut)” (light orange) under indicated feeding conditions. Numbers of fed mosquitoes for each condition are indicated to the right of the graph. (**E**) Pie charts showing fractions of mosquitoes from D scored and color coded as in Fig. 1. (**F**) Distribution scores (see STAR METHODS) for results shown in E. For all experiments, mosquitoes were starved for ~ 24 h with access to water prior to feeding on a membrane feeder with heat.

Nectar contains various sugars^1,45–49^, which are different in nutritional value^50–52^ as well as in their activity on sweet taste neurons^53–55^. To determine if the identity of the sugar solution affects diversion to the crop, we tested 12 different sugars/sweet tastants that fall into four categories based on nutritional value and taste, as determined from work with flies: (1) sweet and nutritive (D-sucrose, D-maltose, D-trehalose, D-fructose and D-glucose), (2) sweet but not nutritive (L-glucose, D-arabinose and sucralose), (3) nutritive but not sweet (D-mannose and D-sorbitol), and (4), neither nutritive nor sweet (D-ribose). Each tastant solution was prepared at a 1 M (except for sucralose at 50 mM) concentration and provided to mosquitoes using the artificial membrane feeder. Mosquitoes were then dissected and scored for the presence of sugar solution in the crop and midgut (Fig. 3A,B,C).

Solutions of all five sweet and nutritive sugars were efficiently diverted to the crop, with < 5.7% of fed animals exhibiting a “midgut only” distribution of the imbibed meal (Figs. 3A; 1 M glucose; 1 M trehalose; 1 M fructose; 1 M sucrose; 1 M maltose, and 3C; 1 M glucose; 1 M maltose [mean distribution score: 0.7626–0.8143]). The proportion of mosquitoes fed on the other solutions and failing to divert the meal to the crop was distributed along a continuum, ranging from 8.97% (arabinose) to 36.6% (mannose) (Fig. 3A; 1 M mannose; 1 M L-glucose; 1 M ribose; 50 mM sucralose; 1 M sorbitol; 1 M arabinose). Mean distribution scores of Fig. 3C; 1 M mannose (1.019); 1 M L-glucose (1.149); 1 M ribose (1.030); 50 mM sucralose (1.000); 1 M sorbitol (0.8602); 1 M arabinose (0.8458). Sugars with low nutritional value and sweetness (mannose and ribose) did not activate the switching mechanism, and the imbibed solution was distributed much like that of water (Figs. 3A; 1 M mannose; 1 M ribose, and 2 A; water (40 °C)). Most of the other solutions elicited intermediate phenotypes, with no clearly separable effects of nutritive value or sweetness. These results indicate that sugar/sweet tastant identity is a factor in directing solutions to the crop, with a higher likelihood for those with higher nutritional value and sweetness.

One caveat is that not all solutions were consumed to the same extent (Fig. 3B). In particular, for solutions with which intake was low, a “midgut only” distribution may have been underscored. We therefore confirmed the effect of individual sugars/sweet tastants on the switching mechanism by testing them in mixtures with blood (Fig. 3D and F). Consistent with the findings in Fig. 3A, we observed that addition of sweet and nutritive sugars (sucrose, maltose, trehalose, D-glucose) diverted the mixture to the crop (Fig. 3D; blood (5 ml) + trehalose (1 g); blood (5 ml) + glucose (1 g); blood (5 ml) + sucrose (1 g); blood (5 ml) + maltose (1 g), Fig. 3E; blood (5 ml) + trehalose (1 g); blood (5 ml) + glucose (1 g); blood (5 ml) + sucrose (1 g), and Fig. 3F; blood (5 ml) + trehalose (1 g); blood (5 ml) + glucose (1 g); blood (5 ml) + sucrose (1 g) [mean distribution score: 1.301–1.680]). Conversely, we note that when blood was mixed with L-glucose, intake was significantly reduced (Fig. 3E; blood (5 ml) + L-glucose (1 g)). As observed in the context of pure solutions, the other sugar/sweet tastants had low to intermediate effects on crop entry even when mixed with blood (Fig. 3D; blood (5 ml) + ribose (1 g); blood (5 ml) + L-glucose (1 g); blood (5 ml) + arabinose (1 g); blood (5 ml) + sorbitol (1 g); blood (5 ml) + mannnose (1 g), and 3 F; blood (5 ml) + L-glucose (1 g), [mean distribution score: 1.375]). Collectively, our results suggest that there are mechanisms whereby blood is actively directed to the midgut and sweet, nutritive sugars are actively directed to the crop.

### Robust meal sorting is necessary for egg production

To determine the functional importance of meal sorting, we fed mosquitoes with sugar-blood mixtures that disrupted proper sorting and examined survival and egg development. First, we mixed 0.1 g, 0.5 g, or 1.0 g of sucrose per 5 mL of blood and gave the mixtures to mosquitoes in an artificial membrane feeder. Inspection of meal destination in fed mosquitoes showed that blood-sugar mixtures were found in the crop. Moreover, the higher the amount of sucrose in the blood, the higher was the amount of “blood” found in the crop (Figs. 1D; starved, blood (40 °C), 4A and 3E; blood (5 ml) + sucrose (1 g)). Thus, the sucrose-blood mixture is diverted to the crop in a sucrose concentration-dependent manner.

**Fig. 4:**
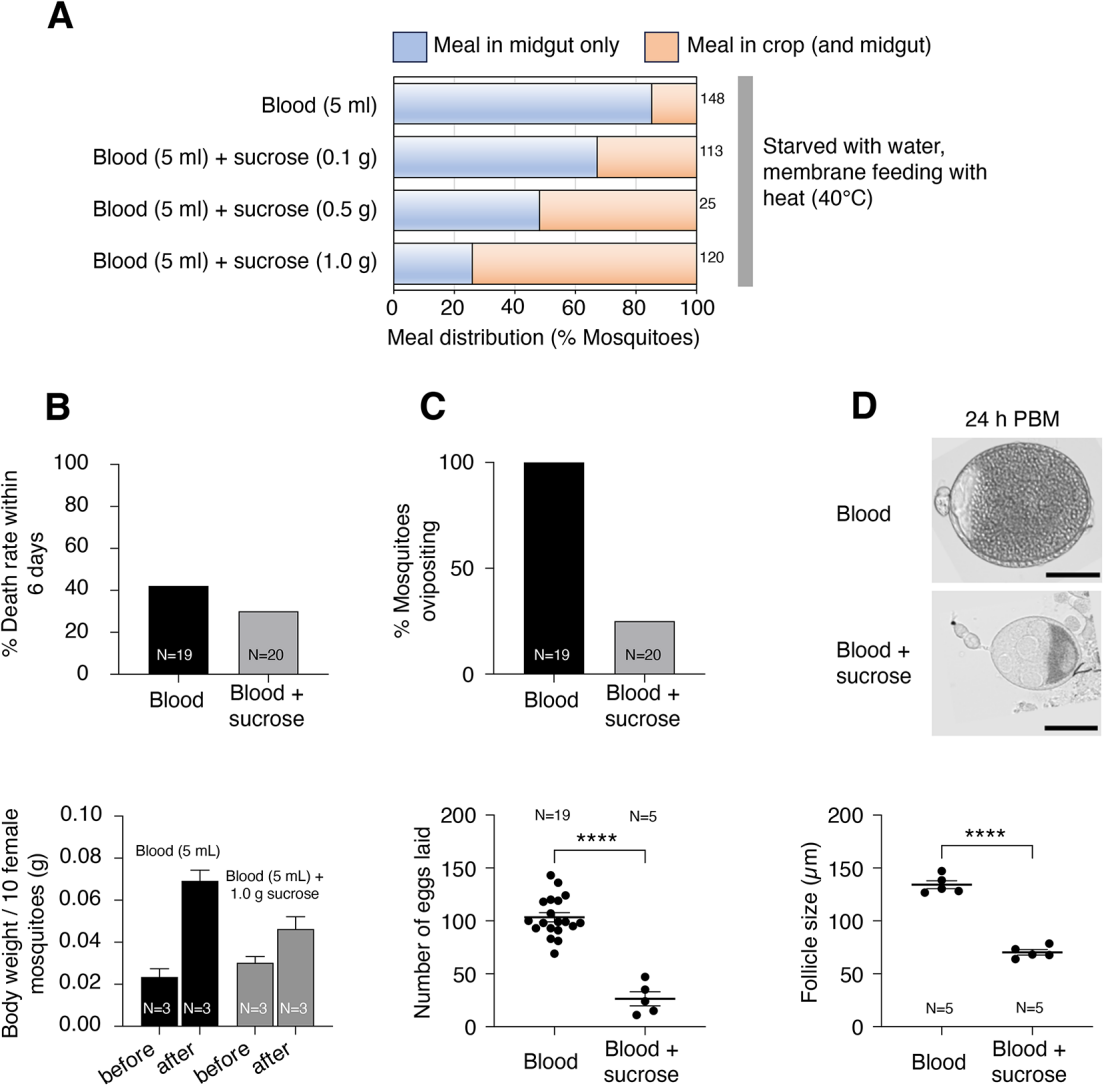
Loosening the crop valve during blood-feeding reduces offspring. (**A**) Percent of mosquitoes showing “meal in midgut only” (light blue) and “meal in crop (and midgut)” (light orange) under indicated feeding conditions. Mosquitoes were starved for ~ 24 h with access to water prior to feeding on a membrane feeder with heat. Numbers of fed mosquitoes for each condition are indicated to the right of the graph. (**B**) Percent mortality in the 6-day period after feeding (top). Body weight of female mosquitoes before and immediately after feeding (bottom). (**C**) Percent of females that laid eggs (top) and mean number of eggs laid per female (bottom). ****p < 0.0001, two-tailed unpaired *t*-test. (**D**) Representative images of primary egg chambers at 24 h (top) and mean follicle size (bottom) after feeding on indicated meal. “blood + sucrose” indicates a mixture of blood (5 ml) and sucrose (1.0 g). ****p < 0.0001, two-tailed unpaired *t*-test. Scale bars = 50 μm.

We elected to test the consequences of imbibing a mixture of blood and 1.0 g of sucrose, the solution that most dramatically disrupted meal sorting. In agreement with the manually assigned meal volume score (Fig. 1D; starved, blood (40 °C), and 3E; blood (5 ml) + sucrose (1 g)) that points to a lower intake of blood-sugar solutions, comparison of body weight before and after feeding indicated an average weight gain of 221.6% after a pure blood meal in contrast with a gain of 52.7% after a blood-sugar meal (Fig. 4B, bottom). There were no indications that disruption of meal sorting, or indeed any other feature of the blood/sugar mixture, had immediate deleterious effects. The percentage of individuals that died within 6 days of feeding was 42.1% for pure blood and 30% for the blood/sucrose mixture (Fig. 4B, top).

We next examined features of egg production. First, all individuals that fed on pure blood laid eggs (100%), whereas only 25% of those that fed on the blood-sugar mixture did so (Fig. 4C, top). Further, even among the egg-laying females, the number of eggs laid by each mosquito that took the blood-sugar meal was also significantly reduced (Fig. 4C, bottom). Follicle (or egg chamber) size measurements taken 24 h after blood feeding revealed significant reductions in blood-sugar-fed females as compared to blood-fed females (Fig. 4D). Notably, the reduction of ovariole size and fecundity appears to be much more dramatic than the reduction observed for meal volume, consistent with the idea that mis-sorting of blood meals may be a contributing factor in the decrease of total egg production.

### Entry of amino acids into the crop depends on their identity

**Fig. 5:**
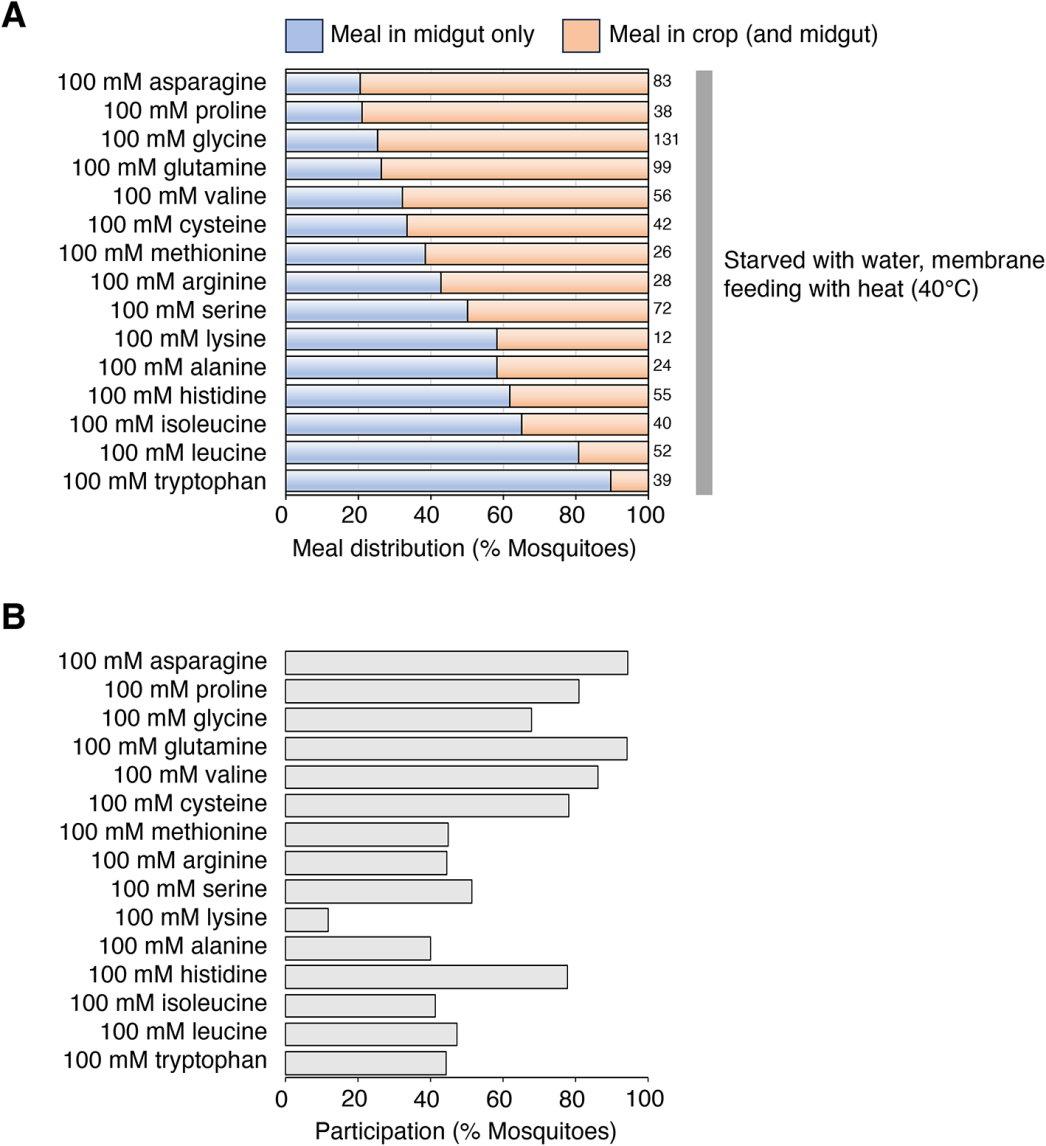
The entry of amino acids into the crop depends on their types. (**A**) Percent of mosquitoes showing “meal in midgut only” (light blue) and “meal in crop (and midgut)” (light orange) with indicated meal solutions. Mosquitoes were starved for ~ 24 h with access to water prior to feeding on a membrane feeder with heat. Numbers of fed mosquitoes for each condition are indicated to the right of the graph. (**B**) Feeding participation of females from A.

Amino acids (AAs) are critical micronutrients, and floral nectar contains small amounts of AAs^44,48,49,56–58^. AAs are also present in blood, both before and after digestion of the blood meal, and a number of them are important for activating vitellogenesis and supporting embryonic development in mosquitoes^4^. Therefore, we examined the meal destination of 15 AA solutions using the artificial membrane feeder. All AAs were dissolved in water and no other chemicals were included. The fraction of fed mosquitoes in which the meal was restricted to the midgut was distributed along a continuum, ranging from 20.5% (asparagine) to 89.7% (tryptophan), representing sugar-like and blood-like phenotypes at the two extremes (Fig. 5A). There was no correlation between the pH value of each AA solution and its meal destination (data not shown). These results suggest that AA sensing can inform the switching mechanism. Of interest is the observation that leucine and tryptophan, the top two AAs found to be essential for activating Vg expression^4^, are diverted in a blood-like manner to the midgut (Fig. 5A; 100 mM leucine, 100 mM tryptophan). Overall, AA ingestion volumes tended to be lower than those of sugar solutions (data not shown). We also found a (weak) correlation between the likelihood of feeding on an AA solution (participation) and the likelihood of crop entry of the imbibed solution, suggesting that there may be a correlation between AA feeding preference and switching (Fig. 5A and B).

## Discussion

**Fig. 6:**
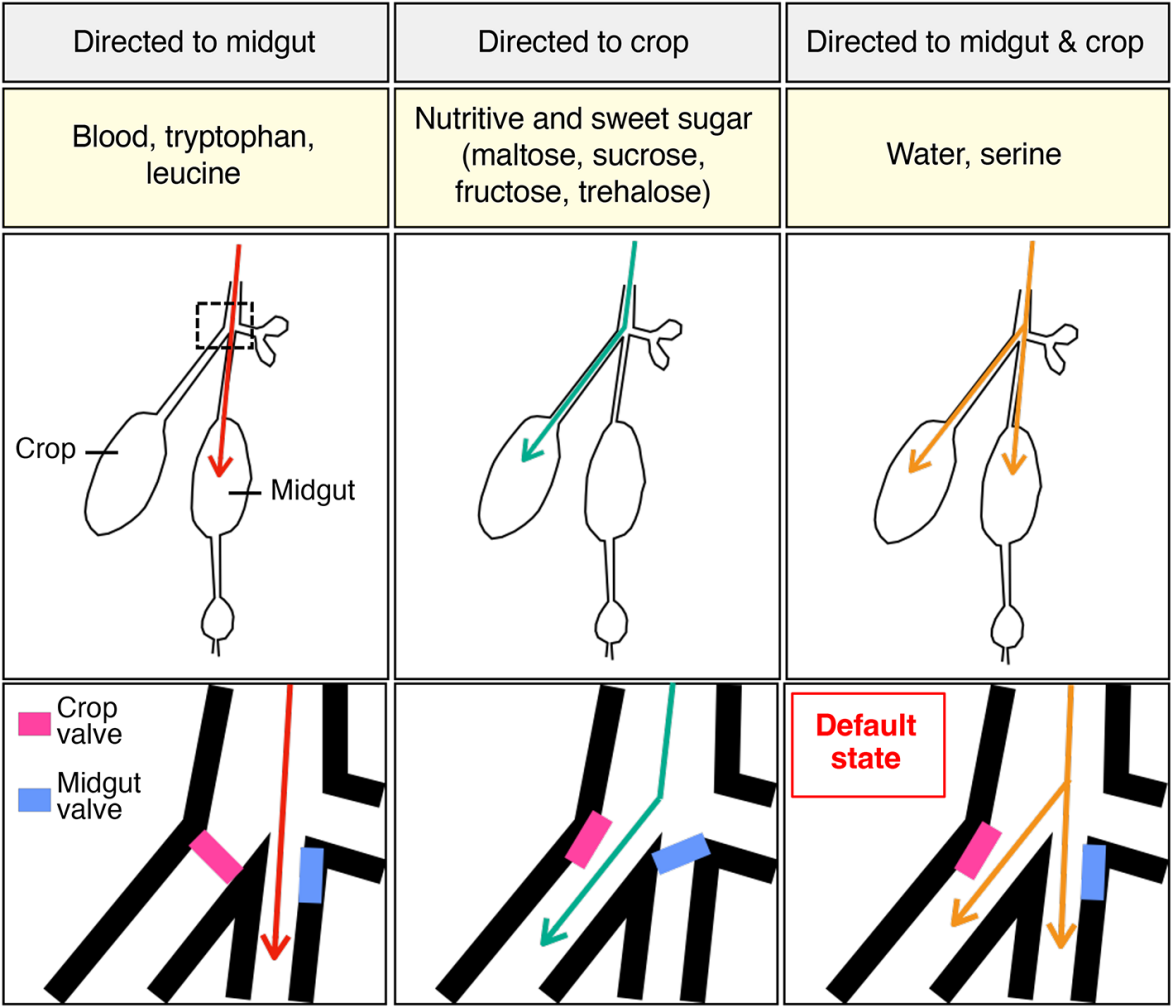
Hypothetical model of meal sorting. Schematic of proposed model of meal sorting to the midgut (left), crop (center) or both midgut and crop (right) indicating examples of sorted meals (based on our results). The potential regulation of crop and midgut valves is diagrammed below.

Here, we characterize meal features that are involved in bringing about robust meal switching. We find that sorting of blood and sugar meals into the midgut and crop, respectively, is influenced by a complex interplay of sensory cues – the most relevant signals are likely to be chemical cues, but temperature and feeding mode also contribute to meal sorting soundness. Consistent with previous studies^30,43^, our results suggest that components in both sugar and blood are sensed by the mosquito to effect meal switching in *Ae. aegypti*. Our observation that ingested water is distributed evenly between the midgut and crop (Fig. 2A; water (40 °C)), is consistent with a model in which both valves are open, at least in part, under default conditions (Fig. 6). In this model, the crop valve would be a primary target of the switching mechanism; detection of blood cues would cause constriction of the valve and direct the ingested blood solely to the midgut. Conversely, detection of a nutritious and sweet sugar solution would cause further opening of the valve and allow most of the ingested solution to enter crop. That proper allocation of nutrients to distinct compartments may be crucial for optimizing reproductive output is suggested by our finding that disrupting the sorting of blood meals by adding sucrose results in reduced egg production and follicle size.

One of our key findings is that blood meals are actively directed to the midgut, and this process is mediated by multiple factors, including blood components, temperature, and feeding mode. The observation that the presence of leukocytes, platelets, and red blood cells is important for diverting blood to the midgut suggests that mosquitoes have evolved mechanisms to detect and respond to the unique composition of blood. However, no single mutation in *Orco*, or *Ir76b* (not shown), co-receptors disrupted the bypassing of the crop during blood feeding, indicating potential redundancies in detection of blood-derived factors. In addition, *Ir7a* and *Ir7f*, expressed in female stylet-specific, blood-responsive neurons, may play a role in closing the crop valve^43^. It will be interesting to determine the underlying mechanisms for sensing relevant blood components. An intriguing possibility is that the presence of disease pathogens in the host may influence meal destination in the mosquito, since differential perturbation of the serum metabolome has been observed following infection with dengue virus^59^.

Moreover, while previous reports concluded that the method of intake had little if any influence on the destination of ingested liquids^8,30,60^, our results found a significant contribution of temperature and feeding mode in controlling blood meal destination (i.e. constricting the crop valve). While our results implicate partial contributions TrpA1 and Gr19, the two are broadly expressed and it’ll be interesting to identify the cells in which their functions are required for meal switching. Similarly, there are several candidate mechanoreceptors, including tmc, Nompc, Piezo, and TMEM63^61–65^, which may respond to the significant force that is exerted by the stylet fascicle during membrane piercing^66^, and future studies will be required to identify which are involved and determine if mechanosensory information from the stylet is conveyed to circuits that control the crop or midgut valves.

In contrast to blood meals, we found that sugar solutions are directed to the crop in a dose-dependent manner, with a preference for sweet and nutritive sugars. This suggests that mosquitoes have the capacity to discriminate between different types of sugars based on their nutritional value and taste. Floral nectar is detected by the labium^67,68^. In our study, sugar solutions were given to the mosquitoes in the artificial feeder, for which the mosquitoes must pierce a membrane to imbibe the solution. Since sugar solutions nevertheless entered the crop, it is likely that the labium is not involved in the sugar detection that triggers switching. Gustatory receptors that are predicted orthologs of *D. melanogaster* canonical sugar receptors, which include Gr4-7, Gr9-11 and Gr14 are not expressed in the stylet that is used for piercing and sucking^43^. Furthermore, blood-responsive neurons in this organ are not activated by sucrose or fructose solutions^43^, although Gr34, the ortholog of the DmGr43a fructose receptor^69^, is expressed in it. Thus, sugar receptors involved in switching (i.e., inducing wider opening of the crop valve) are likely expressed in organs that lie between the stylet and the crop (e.g., cibarial pump, pharyngeal pump, pharynx, esophagus). It may be possible to identify genetic factors that regulate blood meal destination by performing transcriptome analysis of these organs, which could inform development of vector control interventions.

Our findings diverge from the previous reports of the “default” status of the crop and midgut valves in the unfed mosquito as “midgut open, crop closed”, which arose from observations that water and low concentrations of sucrose or cellobiose, when ingested in small quantities, were primarily directed to the midgut^12,35^. One possible reason for the discrepancy is variation in starvation conditions imposed on mosquitoes prior to experiments. In most previous studies, mosquitoes were subjected to pre-starvation periods without water for several hours before testing them in feeding assays^33,70^. Studies involving *Drosophila* have demonstrated that flies exhibit high sensitivity to dehydration during periods of starvation^71^. Recent studies have described significant changes in gene expression between glial cells from water-satiated and water-deprived flies^72^. Moreover, our experiments indicate substantial disruption of the meal switching system when blood was provided after a period of water-deprived starvation (Fig. 1B; starved without water, blood (40 °C)). Taken together, these findings raise the possibility that water-deprived starvation conditions may confound analysis of the switching mechanism due to broad effects on physiology and behavior, and our approach to provide water during starvation to prevent dehydration may have yielded more robust and reliable data. As meal sorting is essential for female mosquito fitness and reproduction, artificial intervention of the meal switching mechanism may serve as an effective means for mosquito control.

## Limitations of the study

While our study provides some important insights into the regulation of meal sorting in *Ae. aegypti* mosquitoes, there are several limitations to consider. First, our experiments were conducted using an artificial membrane feeding system, which may not fully recapitulate the complex sensory and physiological cues present during natural feeding on a host. Future studies could investigate meal sorting in more naturalistic feeding contexts to validate and extend our findings. Second, blood glucose levels vary significantly across animal species^73^. For example, blood glucose levels in birds are two-to-fourfold higher (> 150 mg/dL) than in mammals, with levels up to 800 mg/dL in hummingbirds^74–76^. Thus, further research is needed to determine whether the source of a blood meal impacts its destination in the mosquito. Third, while we identified several factors that influence meal destination, the underlying molecular and neural mechanisms of how different cues are sensed and integrated remain to be elucidated. Our study opens up new avenues for further work to identify the specific receptors, signaling pathways, and neural circuits that mediate the detection and processing of meal-related cues that control the switching mechanism.

## Methods

### Mosquito rearing and maintenance

*Aedes aegypti* wildtype (Orlando) and mutant strains were reared in cages at 28ºC, 70–80% relative humidity under a 12:12 light-dark cycle. Mosquitoes were given constant access to a 10% sucrose solution. Blood-feeding was carried out using an artificial membrane feeder with bovine blood warmed to 39–40 °C using a water-bath. Eggs laid on wet filter papers were transferred to water trays. Larvae were fed TetraMin (Tetra) fish food. *AaegTRPA1* and *AaegGr19*^41^, and *orco*^16^ ^77^ mutants were provided by L.B. Vosshall.

For collecting virgins, individual pupae were housed in vials and sexed upon adult eclosion. Females (*n* = 90–108) were transferred to a cage, given access to 10% sucrose, and tested after 6–8 days.

### Membrane feeding

The artificial feeding assay system was assembled as shown in Fig. 1A, and the bottom of a hand-made glass feeder was covered with stretched Parafilm. 0.5 µg/µl sulforhodamine B (Sigma-Aldrich, 230162) pink dye was included in all tastant solutions except blood, which were dispensed into a feeder, set to 40 °C, that was placed on a cage containing approximately 500 mosquitoes (males and females combined). After ~ 30 min, all mosquitoes were aspirated, females were dissected to extract the gut, and the midgut and crop were observed under a stereomicroscope to visually score the presence or absence of imbibed solution. Individuals with no detectable meal in the midgut and crop were excluded as unfed. Fed animals were categorized according to Fig. 1C.

### Distribution score

The meal distribution score for individual mosquitoes that were dissected and assessed for imbibed solutions in the crop and midgut after various experimental feeding conditions was calculated as follows. Midgut score and crop score were determined as illustrated in Fig. 1C and a distribution score was calculated as follows:

$${\text{Distribution score}} = ({\text{midgut score}} +1)/({\text{crop score}}+1).$$

1 was added to the midgut and crop scores (scored as in Fig. 1C) for computational convenience. A distribution score of 1 would indicate an equal proportion of the meal in the midgut and crop, whereas a score > 1 would indicate a higher proportion of the meal in the midgut.

### Chemicals

Chemicals were obtained from the Sigma-Aldrich: D-(+)-Mannose (M6020), L(-)-Glucose (G5500), D-(-)-Ribose (R7500), Sucralose (J66736, Alfa Aesar), D-(-)-Sorbitol (85529), D-(+)-Fucose (21940, Chem-Impex), D-(-)-Arabinose (A3131), D-(+)-Glucose (G6152), D-(+)-Trehalose (D-(+)-Trehalose dihydrate, T9531), Fructose (47740, Fluka), Sucrose (S7903), D-(+)-Maltose (M9171), L-Asparagine (11149), L-Proline (81709), Glycine (50046), L-Glutamine (G8540), L-Valine (94619), L-Cysteine (30089), L-Methionine (64319), L-Arginine (A8094), L-Serine (84959), L-Lysine (L5501), L-Alanine (05129), L-Histidine (53319), L-Isoleucine (I7403), L-Leucine (61819), L-Tryptophan (93659). Adenosine 5’-triphosphate disodium salt hydrate (ATP) was obtained from TCI (A0157). NaCl was obtained from Macron Chemical (7581-06).

### Death rate and body weight measurement

Female mortality was assessed six days after blood feeding by counting the number of dead mosquitoes. Mosquito weights were determined before and immediately after blood feeding by weighing cohorts of 10 females in 1.5 mL tubes.

### Egg chamber measurement

Twenty-four hours after blood-feeding (24 h PBM), egg chambers were dissected from blood-fed mosquitoes and their lengths (long axis) were measured using an Olympus compound microscope (BX51W1) and MetaMorph software. Five different egg chambers were measured in each mosquito.

### Oviposition rate and egg number

Individual blood-fed mosquitoes showing engorged abdomens were collected and transferred to vials with wetted paper towels for egg deposition and cotton balls soaked with 10% sucrose. Eggs on the paper towel were counted under a stereomicroscope after 4 days (vials with dead females and/or no eggs were excluded). On the other hand, the number of individuals that did not oviposit was counted and included in calculations of oviposition rate.

## Data Availability

All datasets generated during the current study are available from the corresponding author upon request.
